# Treatment of Liver Metastases in Patients with Neuroendocrine Tumors: A Comprehensive Review

**DOI:** 10.4061/2011/154541

**Published:** 2011-10-13

**Authors:** Theresa R. Harring, N. Thao N. Nguyen, John A. Goss, Christine A. O'Mahony

**Affiliations:** ^1^Michael E. DeBakey Department of Surgery, Baylor College of Medicine, One Baylor Plaza, Suite No. 404D, Houston, TX 77030, USA; ^2^Division of Abdominal Transplantation, The Liver Center, Michael E. DeBakey Department of Surgery, Baylor College of Medicine, 1709 Dryden Street, Suite No. 1500, Houston, TX 77030, USA

## Abstract

Patients diagnosed with Neuroendocrine Tumors (NET) often are also diagnosed with Neuroendocrine Liver Metastases (NLM) during the course of their disease. NLM can cause significant morbidity and mortality, oftentimes much more than compared to patients with NET. Treatment options have been limited in the past, focusing on surgical resections, for which only a minority of patients are candidates. However, developments of new treatment modalities have progressed rapidly and patients with NLM now have significantly more options, including surgical-directed therapies; liver-directed therapies; and nonsurgical, non-liver-directed therapies. This review provides information about the roles of hepatic resection, orthotopic liver resection, radiofrequency ablation, hepatic artery embolization and hepatic artery chemoembolization, hepatic artery radioembolization and selective internal radiation therapy, peptide receptor radionuclide therapy, systemic chemotherapy, biotherapies including somatostatin analogs and interferon-**α**, vascular endothelial growth factor and mTOR targets, and microRNA-regulated pathways. Given these new options, the clinician can tailor therapy specific to the patient diagnosed with NLM, thereby giving the patient the best possible chance of prolonged survival.

## 1. Introduction

Patients with Neuroendocrine Tumors (NETs) often suffer from Neuroendocrine Liver Metastases (NLMs) causing significant morbidity and mortality. The excess hormone production, the multitude of hepatic lesions, and ultimate liver disease lend to the poorer prognosis. In fact, 46%–93% of patients with NETs will find NLMs involved at the time of diagnosis [1]. Patients with liver metastases have a significantly worse prognosis than those without liver involvement. The 5-year survival of patients with NLMs on supportive care is 0%–20% [1–3]. This dismal prognosis paints a much more stark reality for a pathological process often described as “indolent”. Surgical interventions for NLMs have consistently been shown to have superior outcomes to nonoperative therapies. Resection alone is supported by favorable long-term outcomes in large retrospective trials [2]; however, complete surgical extirpation is an option for a very small percentage of the neuroendocrine cancer patient population [1]. Due to excessive metastatic tumor burden in difficult locations, surgical resections are limited to only 10% of these patients [2]. Treatment options for patients that are not surgical candidates have evolved over the last several years. Use of ablative techniques, as well as development of new medical therapies, has expanded the treatment options for the majority of patients with NLMs.

## 2. Surgical-Directed Therapies

Surgery remains the only potential for cure in patients with NLMs. Even in the setting of incurable disease, surgery offers the best chance for prolonged survival. In patients treated with resection, the five-year survival has been shown to be greater than 60% [4, 5] and even approaches 80% in some studies, with minimal mortality (<5%) and morbidity (<30%) [6]. A precise review of the literature available on patients who undergo liver resection for neuroendocrine tumors is difficult due to the small number of patients who are candidates and the varied approaches to surgical treatment [7]. Historically, patients were selected to undergo palliative resection if greater that 90% of the tumor burden could be excised [8]. One of the earlier prospective studies concluded from their study of 47 patients that hepatic resection is indicated only when all gross disease can be removed safely. In this study, they determined that number, size, and location of primary tumor were less important than the completeness of resection. Patients that underwent a complete resection had a 5-year survival of 80%. However, the patients that underwent an R1 resection had a 5-year survival of 70% and R2 resection still had a 5-year survival of 60%. Although patients in this study were included only if it appeared that they could be completely resected, patients that had incomplete resection still did well [9]. Several other series have reported similar results [4, 6]. A more recent retrospective review of 74 cases demonstrated a greater than 60% 5-year survival in all patients that underwent resection [10]. Only 65% of these patients had all gross disease completely excised.

Although an aggressive surgical approach is considered to prolong survival and contribute to better symptom control, the criteria for patient selection are ill defined. In an effort to identify variables that have prognostic relevance to patients who undergo hepatic resection, a prospective review of 70 patients' outcomes was performed based on tumor grade. The tumors were categorized as low grade (<2 mitotic figures/50 hpf and no necrosis), intermediate grade (2–50 mitotic figures/50 hpf and/or focal necrosis), and high grade (>50 mitotic figures/50 hpf and/or extensive necrosis). The majority of the neoplasms were considered low grade (37) or intermediate grade (26). Only 7 were shown to be high grade. The overall 5-year survival rate was 61%. None of the patients with high-grade malignancy survived 5 years with a median survival of only 6 months [7]. The importance of tumor grade to patient's outcomes after resection has been confirmed by several investigations [10, 11]. 

Tumor size, number, and location have also been shown to influence postresection survival [4, 12]. In 2008, the ENETS proposed guideline for surgical resection based on the 3 distinct patterns of liver involvement: (1) “simple” pattern of metastasis located in one or two contiguous lobes (20–25%), (2) “complex” pattern where there is one major focus and other lesions are contained in the contralateral lobe (10–15%), and (3) “diffuse” disease in both lobes (60–70%) [13]. The type of surgical resection is based on the patient's overall medical condition, size, number, and location of lesions, and adequacy of remnant liver size/function. In patients with the simple pattern of disease, an anatomic resection is adequate to completely resect all disease. Patients with the complex pattern of disease can be treated with several different methods. An anatomic lobectomy can be performed for the majority of the disease and either a wedge resection or locally ablative therapy can treat the remainder of the tumors. Staged, multiple surgical procedures have also been shown to be beneficial with little increase in morbidity and mortality [13]. 

The majority of patients with “diffuse” disease are not candidates for resection. Cytoreductive surgery can be helpful for a small, select group of patients. It is usually recommended only in cases where >90% of the tumor volume can be excised or in very young patients [12]. In patients that are symptomatic, cytoreductive surgery has been shown to improve or alleviate their symptoms for a prolonged period of time. In addition, tumor debulking may also increase the effectiveness of medical therapy. 

It has been estimated that less than 20% of patients with metastatic neuroendocrine tumors are candidates for hepatic resection [14]. Resection is not a viable option for the majority of patients with diffuse hepatic disease. Based on their slow growth and good response to resection, liver transplantation has been tried in an attempt to cure, prolong survival or control symptoms. Although many centers are reluctant to allocate liver allografts to patients with metastatic disease, liver transplantation for neuroendocrine tumors is one of the only accepted indications for transplant in the setting of metastatic disease. In 1998, Lehnert analyzed a total of 103 patients transplanted for metastatic neuroendocrine carcinoma. The overall 5-year survival was 47%, and disease-free survival was 24%. Tumor histology or location of primary did not appear to effect survival in this study. However, extent of surgery at the time of transplantation and age of recipient were significant prognostic factors for survival [15]. A more recent retrospective study was performed in 2008. 85 patients were identified who underwent OLT for metastatic neuroendocrine carcinoma in France. The overall 5-year survival was comparable at 47% and disease-free survival of 20% at 5 years. In this study, primary tumor location in the duodenum or pancreas was noted to be an indicator of poor prognosis [16]. This finding was not supported in several other investigations [2, 17]. One of the larger single center studies attempted to analyze tumor biology in relation to postliver transplant outcomes. These authors studied Ki-67, E-Cadherin, and p53. Based on evaluation of 19 cases, they demonstrated that patients with a low Ki-67 (<5%) and normal E-Cadherin staining did significantly better than patients with high Ki-67 or abnormal E-Cadherin expression. Expression of p53 did not appear to influence survival [18]. 

 Analysis of the United Network for Organ Sharing database reveals that between November 1988 and March 2011, only 185 liver transplants were performed for metastatic neuroendocrine tumors in the United States. The overall 5-year survival was 57.8%. This is significantly worse than the 74% 5-year survival for all other patients. Although the long-term survival is not comparable to other patients with benign disease, most liver transplant programs will consider evaluating patients with NLMs. Many liver transplant programs will consider a patient with metastatic neuroendocrine for liver transplantation if the following criteria are met:

1. not a resection candidate,
2. identification and complete resection of primary malignancy at least one year prior to evaluation,
3. no evidence of extrahepatic disease demonstrated on cross-sectional imaging or nuclear medicine scan,
4. evidence of stability of disease for at least one year,
5. failure of nonoperative treatments.

Liver transplantation for metastatic neuroendocrine tumors remains controversial. This radical treatment occasionally provides a cure, but the long-term survival is still significantly less than in patients transplanted for other diseases. It can prolong survival and provide symptomatic relief in a very small subset of patients. Patients that are younger than 50 years in the setting of low Ki-67 and E-Cadherin expression with symptoms that are difficult to control appear to benefit the most from liver transplantation. 

## 3. Liver-Directed Therapies

NETs are predisposed to form highly vascular metastatic lesions in the liver and derive more than 90% of their oxygenation and nutrition from the hepatic artery. Thus, the hepatic artery offers a viable mode of introducing directed chemotherapy and/or creating an ischemic environment. This effectively starves the tumors of their nutrient and oxygen supply while sparing healthy hepatic cells, which derive the majority of their nutrient and oxygen supply from the portal venous system. Several ablative techniques have been developed that exploit the dual blood supply of the liver in an effort to control the disease process.

Defining the treatment best suited for the tumor load is dependent on number and location of the lesions, invasiveness and size of the tumor, physiology and effects of hormone secretion, and extent of metastatic disease within the patient. This is in conjunction with the ultimate goals of cure or palliation. Considering the rate of recurrence, liver-directed therapies have been considered more as debulking modalities. In a review of the literature, general guidelines for the treatment pathways are: for fewer nodular liver metastatic lesions, local resection or thermal ablation is recommended; for a higher-tumor load due to unresectable multinodular disease or recurrent disease after resection, hepatic artery embolization, hepatic artery chemoembolization, or radioembolization is warranted [19]. These modalities are also useful as “neoadjuvants” to decrease the size of previously unresectable metastatic disease. Unless 80%–90% of the tumor load is debulked, treatment does not serve useful as palliation therapy to prolong survival and improve symptom control [20]. 

### 3.1. Radiofrequency Ablation

Radiofrequency ablation (RFA) uses an image-guided technique, percutaneous, laparoscopic, or open, to provide local control with short-term symptomatic relief [21] by subjecting tumors to intense, destructive heat using an alternating electric current. This technique is amenable to patients with fewer liver metastases who are ineligible for hepatic resection. It is used as a single modality, often more than once, or as an adjunct to other NLMs therapies for debulking.

The largest study to date, with the longest followup, was done at the Cleveland Clinic [21], a prospective trial of 89 patients with NLMs who underwent 119 laparoscopic RFA sessions in total. Ninety-seven percent of the sample immediately felt improvement of symptoms after the procedure, where median disease-free survival was 1.3 years and overall survival at 6 years after RFA [21]. Of note, 22% of this sample developed local recurrence, with 63% developing new lesions and 59% developing extrahepatic disease.

Prior to that study, Mazzaglia et al. investigated a series of 63 patients who had a total of 452 treated NLMs lesions. Symptoms were controlled an average of 11 ± 2.3 months after RFA, with greater than 90% of symptomatic patients experiencing relief immediately after procedure. Mean survival extended 3.9 years after the first RFA treatment. Larger dominant tumor size (>3 cm) and male sex were significant variables negatively correlated with survival [22].

A United Kingdom group describes RFA of 189 lesions in 25 patients. Median survival of the group was 53 months from liver diagnosis. Of those with radiologic followup, 74% of patients were noted to have tumor load control at a median of 21 months after the procedure. This meant complete, partial, or static tumor response to RFA. Hormonal treatment has also been used as an adjunct to improve symptomatic relief, though not improve survival. Adjuvant octreotide has been shown to extend median symptom-free duration from 16 to 60 months [19]. It has not, however, been proven to increase survival.

Morbidity for radiofrequency ablation of liver metastases has been reported in the larger studies to be approximately 5% to 12%, with 30-day mortality at 0% to 1%. [21, 22] The complications can include carcinoid crisis, liver abscesses, biliopleural fistulas, bile leakage, and pleural effusion, as well as postablation syndrome, and liver failure.

### 3.2. Hepatic Artery Embolization and Hepatic Artery Chemoembolization

Capitalizing on the dual blood supply of the liver enables a transarterial approach to the hepatic lesions of neuroendocrine metastases. Hepatic artery embolization (HAE) induces ischemia within the tumor, using a variety of agents such as cyanoacrylate, gel foam particles, polyvinyl alcohol, and microspheres. Indications for HAE or hepatic artery chemoembolization (HACE) generally include unresectability with symptoms related to tumor bulk, excessive hormone production, and rapid progression of liver disease [3]. HAE has been shown to improve biophysical markers, palliate symptoms and reduce tumor burden by radiographic evaluation [2, 23]. Because of the observation that higher disease regression rate and longer length of regression with systemic chemotherapy after HAE was published [24], chemotherapy has been added to the embolic agents, and HACE is now generally favored over HAE. HACE, also known as transarterial chemoembolization, combines the hepatic artery embolization with the hepatic artery chemoinfusion where the microspheres are bound to chemotherapy agents, which are then injected into the hepatic artery to lodge downstream within capillaries. Not only do the emboli block the blood supply causing ischemic necrosis, but the chemotherapy agents are localized within the region of the metastatic lesions, creating a much more concentrated effect (up to 20 times greater) than systemic chemotherapy alone [22] as well. Despite this theoretical advantage, little evidence has suggested a significant difference in the outcomes of hepatic artery embolization versus hepatic artery chemoembolization. In a review of the literature, HACE has shown a 5-year survival between 50% to 65% whereas HAE has a 5-year survival between 40% to 67% [24]. In one study of 100 patients with NLMs who received HACE or HAE, the authors found no difference in overall survival, median survival after diagnosis of metastatic disease, or median survival after first embolization [25]. On univariate analysis, the only predictor that significantly improved survival was concurrent resection of the primary tumor, which increased median survival from 28.0 months to 73.1 months [25]. Contrary to this study, Ho et al. reported results on 46 patients with NLMs who received HACE or HAE, and showed that there was no statistically significant survival benefit in a small subset of population that also had resection of the primary tumor, although mean survival after resection increased by a mean of 558 days [26]. Regardless, these therapies have increased versatility as reflected in a study of 48 patients and 123 treatment sessions which revealed HACE or HAE could even benefit carefully selected patients with a tumor load of greater than 75% liver involvement, so long as the patients did not have additional risk factors [27]. Having said this, a number of reports reveal worse outcomes for patients with greater than 50% liver involvement [28, 29]. This is tempered by the fact that extent of liver involvement did not serve as an independent prognostic indicator [3]. In order to mediate the complications arising from disease which takes up the bulk of the liver, it is recommended to divvy small portions of the liver for treatment during each session.

There are adjuvants to HACE or HAE in those patients with severely limited therapeutic options to improve otherwise bleak outcomes, and HACE or HAE can be used as adjuvant therapy to other treatments. Adding hepatic artery chemoinfusion (HAI) to HACE offers an increased probability of clinical benefits to those with unresectable, refractory disease, as presented in a study of 77 patients [30]. The response rate was 80% of islet and carcinoid tumors with a median progression-free survival of 19 months. 1- and 5-year survival rates were 78% and 27% [31]. Of the different types of neuroendocrine tumors, carcinoid tumors seem to consistently have better outcomes to the combination of HACE and HAI [21, 23, 30]. Although studies on patients with NLMs are limited, in one study of 32 patients with hepatocellular carcinoma, the authors found that there was no survival advantage in patients with preoperative HACE prior to surgical resection [32]. In fact, the recurrence-free survival rates were statistically higher, and cumulative recurrence rates were statistically lower at 1, 2, and 5 years compared between the two groups [32]. One study from Iowa on patients with NLMs showed that preoperative HACE followed by OLT can result favorably for the patients with progression-free intervals up to 29 months, but this was a small study, and statistical inferences could not be made due to the inclusion of only four patients [33]. Along a different treatment strategy, Hao et al. showed that survival improved when patients with hepatocellular carcinoma received combination therapy with HACE plus thalidomide versus HACE alone [34]. This improvement reached statistically significant improvement, resulting in median overall survival increases of 15 months [34]. Similarly, in an experimental model utilizing liver tumors in rabbits, favorable outcomes resulting in significantly decreased vascular endothelial growth factor and microvascular density levels were achieved when HACE plus antiangiogenic therapies were used [35]. However, tumor size was not significantly different between these two groups [35].

An important point of HACE or HAE is that response can be incomplete as the periphery of the tumor is spared from ischemia or chemotherapy. With proximal embolization of arterial branches feeding the tumors, peripheral hepatic collaterals reconstitute quickly, requiring repeated embolizations to complete the necrotic process [24]. Multiple sessions are usually needed.

As all other procedures, there are risks involved with liver-directed therapy through the hepatic artery. Liver abscesses, transient liver failure with or without encephalopathy, carcinoid crisis, pleural effusions, and postembolization syndrome (i.e., fever, abdominal pain, leukocytosis, and transient increases in hepatic enzymes and bilirubin) are some of the more common and worrisome. Relative contraindications for these procedures include coagulopathy, renal failure, portal vein occlusion, and liver failure.

### 3.3. Hepatic Artery Radioembolization and Selective Internal Radiation Therapy

Limited effective strategies exist for the treatment of inoperable, refractory NLMs. Interest in one particular liver-directed therapy is under further investigation for this indication: hepatic artery embolization (HAR), also known as selective internal radiation therapy (SIRT). SIRT acts by delivering microspheres of glass or resin, labeled by ^90^Yttrium (^90^Y) to deliver radiation directly into the hepatic artery. Rather than using peptides to localize the lesions, this therapy mechanically targets the metastases and lodges within the nutrient-supplying capillaries, thereby delivering radiation therapy. While this modality has been tested in a limited number of NETs patients, the results thus far have shown promise [31, 36–38]. 

Saxena et al. have been investigating the safety and efficacy of treatment with ^90^Y radioactive microspheres for patients with unresectable NLMs. In this study, 34 such patients were treated with SIRT to achieve long-term responses with a mean overall survival of 29.4 ± 3.4 months, and radiological improvement in 50%. Biochemical marker levels of chromogranin A fell in nearly 50% of survivors by 30 months [31]. 

In one multicenter retrospective review by Kennedy et al., 148 patients with NLMs were followed after radioembolization with ^90^Y [38]. This study reports favorable results with radiological response in 63.2% of patients, stable disease in 22.7%, and progression of disease in only 4.9% [38]. The authors state that one of the largest benefits of this treatment is the stabilization of extensive disease allowing for longer survival periods [38].

Another recent publication investigated 48 patients who underwent similar treatment [37]. Radiographic and serology studies revealed median survival of 35 months with a followup of 41 months, and 55% of patients had complete or partial responses [37]. Less than a quarter of the sample had progressive disease [37]. Prognostic factors were assessed, and 6 of significance were found to influence survivorship: complete/partial response, low hepatic tumor burden, female gender, well-differentiated tumors, and absence of extrahepatic metastases [37]. This was important in identifying a subset of the NLMs patient population who would be best served by this newer technique.

The complications of radioembolization include abdominal pain, nausea, and fever. Radiation gastritis and duodenal ulcers have been described, and as all liver-directed therapies, the risk of liver failure is present. Of note, this promising modality of care, while approved for treatment of colonic cancer metastases to the liver, is still under FDA investigation for treatment of NLMs. Current literature suggests there is significant potential in SIRT/HAR as part of the armamentarium against neuroendocrine tumors and its hepatic metastases.

## 4. Nonsurgical, Non-Liver-Directed Therapies

Since NLMs is a rare disease, large-scale, randomized trials prove difficult, and although these therapies have been used in the treatment of NETs, not all have been specifically used in the treatment of NLMs. Due to the multiple therapies available, the effectiveness of one versus another is difficult to study, and many times nonsurgical, non-liver-directed therapies tend to be lumped together in studies that are available. Moreover, there continues to be a lack of consensus on a nonsurgical treatment algorithm; however, most agree that nonsurgical, non-liver-directed treatments of NETs and NLMs constitute palliative care. At least, one single-center study in the medical literature [12] has proven that aggressive treatment of NLMs with nonsurgical therapy can extend 3- and 5-year survival rates in patients to 76.4% and 63.9% as compared to previously stated survival rates of 39% [14] and 25% [39], respectively. With these encouraging results and the boom in treatment advancements, the older perspective of “wait-and-watch” treatment is considered antiquated. 

### 4.1. Peptide Receptor Radionuclide Therapy

Peptide receptor radionuclide therapy (PRRT) is an upcoming option with enticing advantages, most useful in symptomatic patients with somatostatin receptor-positive tumors, who are not surgical candidates. Between 80% to 95% of gastroenteropancreatic, NETs express somatostatin receptors [40] as demonstrated by ^111^In-pentetreotide scans (OctreoScan, Covidien-Mallinckrodt Imaging, Hazelwood, MO 63042) [41], so PRRT may be useful for a large percentage of NLMs, perhaps in up to 25% of patients [12]. PRRT utilizes the targeting of a molecule to specific receptors located on the surface of tumor cells. Once the molecule interacts with the receptor, it is internalized, thereby delivering specific and localized radiotherapy. This technique allows precise destruction of tumor cells [42, 43], with little interference of nontumor tissue, except for some exposure of renal, bladder, and bone marrow tissues [44]. ^90^Y, ^177^Lutetium (^177^Lu), or ^111^Indium (^111^In) are radionuclides that are linked with a somatostatin analog: octreotide, octreotate, or lanreotide. The more the tumor expresses somatostatin receptors as compared to the surrounding tissue, the more effective the PRRT will be. Somatostatin scintigraphy can predict the effectiveness of PRRT: low uptake indicates 20% chance of effect on liver metastases, whereas high uptake indicates a 60% chance [45]. ^177^Lu-DOTA^0^Tyr^3^octreotate seems to be the most effective PRRT, with a tumor response rate of 35% and tumor stabilization of 80% to 90% of NETs [44], versus ^90^Y-DOTA^0^Tyr^3^octreotide with a tumor response of 4% and tumor stabilization of 70% [46]. After therapy with ^90^Y-DOTA^0^Tyr^3^octreotide or ^177^Lu-DOTA^0^Tyr^3^octreotate, median duration of results were 30 months and 36 months, respectively [45]. In one study with 310 patients, median overall survival rate from initiation of PRRT was 46 months [47]. Further, patients experiencing benefit after one round of PRRT who develop recurrent or progressive disease may benefit from a second round of PRRT [48].

Side effects of PRRT are rare and usually mild consisting most commonly of nausea and vomiting occurring within 24 hours of administration [41], and although anemia and transient toxicity grade 1 have been reported [12], long-lasting adverse side effects are extremely rare. Patients that seem to benefit the most from PRRT have strong radiotracer uptake on OctreoScan, at least as much as the liver [41]. Newer positron emission tomography (PET) imaging platforms such as ^68^Gallium-DOTA^0^Tyr^3^octreotide-PET and ^68^Gallium-DOTA^0^Tyr^3^octreotate-PET are increasingly used to evaluate tumors as they are even more sensitive to radiotracer uptake [40] and may be able to better predict responsiveness to PRRT [49]. Unfortunately, PRRT is not available in the United States until September 2011, when the first clinical trial will begin (http://clinicaltrials.gov/; Identifier: NCT01237457).

### 4.2. Chemotherapy

The use of systemic chemotherapy is less clear in the treatment of NLMs. Several chemotherapeutic agents have been used in multiple trials, but mainly in the study of NETs only, with limited success and restrictions from side effects and toxicities.

The usefulness of chemotherapy in the treatment of NETs seems to be related to primary tumor location and tumor grade [41]. Pancreatic NETs have been treated successfully with nitrosurea streptozocin (STZ) [41]. The greatest efficacy seems to be related to the use of STZ with other chemotherapy agents, including 5-fluorouracil and doxorubicin, but still only results in a median response time of 9.3 months [50]. Dacarbazine (DTIC) is another chemotherapy agent with proven effectiveness in pancreatic NETs, and in one phase II trial demonstrated a response rate of 34% [51]. The alkylating agent, temozolomide, has also shown promise in pancreatic NETs: a phase II study using temozolomide and thalidomide showed a response rate of 45% [52], and a retrospective study of temozolomide and capecitabine showed a response rate of 70%, a median PFS of 18 months, and an overall 2-year survival of 92% [53]. Platinum-based chemotherapy regimens may be useful in patients with high-grade, poorly differentiated NETs, with response rates of 42% to 80% with the use of cisplatin and etoposide [54–56], and 78% with use of oxaliplatin-based regimens [57]. Even with increased response rates, median survival times are of short duration of 8 to 11 months [56]. Therefore, chemotherapy can be used as salvage treatment, but is generally not considered as first-line, nonsurgical treatment. Moreover, the presence of NLMs may be related to worse response to chemotherapy as compared to NETs [58].

### 4.3. Biotherapy

#### 4.3.1. Somatostatin Analogs

Somatostatin exerts its affect by integration with one of five somatostatin receptors, ssts_1-5_ [59], but due to a half-life of only two minutes [60], somatostatin analogs (SSA) have been developed. Newer formulations may be even easier to administer to patients due to a longer half-life of approximately two hours [61].

The principle use of SSA is in the symptomatic relief of NETs and NLMs, although it may be useful for other indications. The use of SSA produces a median biochemical response rate between 0% to 77%; and biochemical and radiographic tumor stability of 28% and 55%, respectively [61–71]. One review article found symptomatic and tumor response to octreotide, octreotide long-acting repeatable (LAR), lanreotide, and lanreotide slow-release depot (autogel) in 74.2%, 77.3%, 63.0%, and 67.5%, and in 57.4%, 69.8%, 46.6%, and 64.4%, respectively [72]. Another investigation demonstrated relief from flushing and diarrhea in 88% of patients after octreotide administration [61]. Interim data from the PROMID study with metastatic midgut NETs, showed a 66% reduction in the risk of disease progression and arrested tumor growth in 69% for a median of 14.3 months [73]. However, over 75% of patients in this study had limited liver involvement of 10% or less, and the response was highest in patients with relatively low tumor burden [73]. The greatest response rates have been witnessed with octreotide doses of 30 mg/day or greater or with lanreotide doses of 5 mg/day or greater [74]; octreotide doses greater than 60 mg/day likely do not have additive effect due to oversaturation of receptor sites [75]. Similar to PRRT, the level of uptake on somatostatin scintigraphy may be an indicator of patient's response to SSA therapy [44]. 

The newest SSA, pasireotide, is still in clinical development stages, but is promising due to binding of ssts_1_, ssts_2_, ssts_3_, and ssts_5_ [41], as compared to octreotide and lanreotide which bind to ssts_2_ and ssts_5_ only. Preliminary data indicate that pasireotide may be useful in patients with symptoms refractory to octreotide, possibly controlling symptoms in up to 27% of these patients [76].

Side effects are infrequent, but nausea, stomach cramping or discomfort, diarrhea, steatorrhea, cardiac abnormalities and arrhythmias, hypothyroidism, and hypoglycemia may occur [40, 41, 77]. Cholelithiasis may arise in up to 50% of patients due to inhibition of gallbladder contractility [41], but only a handful will develop symptoms requiring cholecystectomy [78]. 

#### 4.3.2. Interferon-*α*

Interferons have multiple antitumor effects [79], and they may upregulate somatostatin receptors in NETs [80], thereby providing a useful combination therapeutic option. Interferon-*α* can ameliorate symptoms in 30% to 70% of patients [81, 82], and in some studies has shown promising results with tumor response rate or stabilization in up to 70% of patients [82]. However, the results of three randomized clinical trials involving interferon-*α* and octreotide have mixed results. Two demonstrated increased 5-year survival rate [70] and median survival time [83] in the combination group versus the octreotide-only group, 57% versus 37% and 51 months versus 35 months, respectively; but another trial showed minimal response rates [84]. 

The side effect profile of interferons may preclude wide utilization. Interferon-*α* can cause fevers, chills, myalgias, depression, and myelosuppression [41], and is considered inferior to SSA. However, in patients with progressive disease, combination therapy may be a viable option [85].

Others have examined the role of dopamine receptors and interferon-*β* [86] as other possible targets, but currently, neither of these targets seems promising at this time due to ineffectiveness and short half-life.

### 4.4. Newer Therapies

Patients who have exhausted other therapies may find acceptable treatment through the use of newer treatment strategies. These interventions remain in the investigative process, including targeting vascular endothelial growth factors (VEGF), mTOR pathways, other growth factor receptors, antiproliferative factors, and antiangiogenic factors. Monoclonal antibodies against insulin-like growth factor-1 receptor (IGF-1R): AMG479, IMC-A12, and MK-0646, are currently in clinical phase II studies in patients with metastatic NETs (http://clinicaltrials.gov/, identifier: NCT01024387, NCT00781911, NCT00610129). Others are looking at genetic copy number alterations of tumor suppressor genes [87] and the detection and characterization of circulating tumor cells to reduce metastatic burden [88] as other possible avenues to treat NETs and NLMs.

#### 4.4.1. Targeting Vascular Endothelial Growth Factors

NETs and NLMs frequently overexpress the vascular endothelial growth factor (VEGF) ligand and receptor (VEGFR) [89]. Tumor progression of NETs has also been associated with circulating levels of VEGF [41], therefore VEGF and VEGFR are promising targets. 

In a study where patients on octreotide therapy were randomized into either treatment with bevacizumab, a humanized monoclonal antibody against VEGF, or interferon-*α*, 95% of patients receiving bevacizumab were progression-free after 18 weeks, compared to 67% of patients receiving interferon-*α* [36]. Bevacizumab is associated with reduction of tumor blood flow and longer progression-free survival (PFS) when compared to alternative treatments [36]. Currently, multiple clinical trials of bevacizumab are ongoing (http://clinicaltrials.gov/, identifiers: NCT00569127, NCT00137774, NCT00398320, NCT00227617, NCT00607113). Bevacizumab may cause hypertension and proteinuria [44], so optimal patient selection prior to treatment is mandatory. 

Sunitinib is a tyrosine kinase receptor inhibitor currently approved in the treatment of renal cell carcinoma and gastrointestinal stromal tumors and inhibits VEGFR1, VEGFR2, and VEGFR3. Phase III trials resulted in median PFS of 11.1 months for patients on sunitinib versus 5.5 months for patients receiving placebo (*P* < 0.001) [40, 90, 91]. In Europe, sunitinib is approved for the treatment of unresectable or metastatic, well-differentiated pancreatic NETs with disease progression in adults [40]. Side effects of sunitinib include fatigue, asthenia, diarrhea, nausea, vomiting, anorexia, bleeding complications, mucosal inflammation, hypertension, anemia, granulocytopenia, thrombocytopenia, and hypothyroidism [40].

#### 4.4.2. Targeting mTOR Pathway

The mammalian target of rapamycin (mTOR) pathway is central to the control of cell growth, protein synthesis, and apoptosis and is activated in NETs [40]. Two mTOR inhibitors have been developed and approved for use in renal cell carcinomas [92], everolimus, and temsirolimus, and have been studied in NETs [41, 93–95]. Everolimus has a potential in conjunction with octreotide LAR [93] and as a monotherapeutic agent with a response rate of 20%, a median PFS between 11 and 16 months in three separate phase III trials [41, 96], and with stabilization of disease in 70% with low- to-intermediate grade NETs [93]. Side effects of everolimus include stomatitis, rash, diarrhea, fatigue, infections, noninfectious pneumonitis, anemia, lymphopenia, hypercholesterolemia, hyperlipidemia, and hyperglycemia [40].

### 4.5. MicroRNA-Regulated Pathways

MicroRNAs are small, noncoding RNAs that can function as gene regulators by posttranscriptional processes, such as inducing mRNA degradation or repression of translation [97–100]. MicroRNAs are usually downregulated in cancers [97–100] and have been studied for possible therapeutic interventions. One study identified microRNA-133a, -145, -146, -222, and -106 to be important in primary NETs, whereas microRNA-183, -488, -19a+b were found to be important in metastatic NETs [97]. Further, the same group determined that decreasing levels of microRNA-133a has an important role in the development, progression, and possible metastasis of midgut carcinoid tumors [97]. A different study identified microRNA-142-3p, -142-5p, -155, -146a, and -483 as up-regulated in pancreatic NETs as compared to normal tissue [101]. This study also found that microRNA-210, -431, and -424 were up-regulated in metastases as compared with tumors, suggesting that certain microRNAs could be used to predict the probability of metastasis [101]. Another study showed that anti-microRNA-182 targeting had a therapeutic effect against melanoma liver metastasis, which may be extended to other tumors [102].

 Additional studies are warranted in this area pertaining to microRNA-regulated pathways, but already possible therapeutic targets have been identified by researchers including the high-mobility group A proteins, HMGA1, HMGA2, and the microRNA family let-7 [103, 104]. These targets will be useful as better strategies evolve to care for patients with NLMs and extend their survival. 

## 5. Conclusion

**Figure 1:**
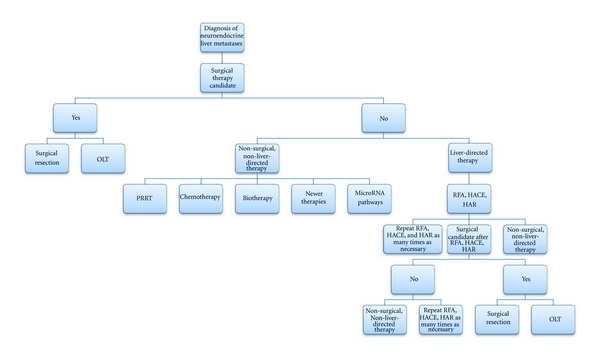
Algorithm for treatment of neuroendocrine liver metastases. The preferred treatment options involve surgical management, followed by liver-directed therapies, or a combination of these procedures. Nonsurgical, non-liver-directed therapies constitute palliative care.

The treatment modalities available to a patient diagnosed with liver metastases due to NETs are vast. The options range from surgical treatments, to locally liver-directed therapies, to systemic approaches (Figure 1). However, most, if not all clinicians, agree that the treatment must be tailored specifically to the patient. Generally, surgical therapies are preferred as they can give the longest disease-free interval. Yet, not all patients with NLMs are candidates for surgical therapy, and in the case of an elderly asymptomatic patient with a slow-growing NETs, the patient may not desire surgical therapy. Liver-directed treatment can also produce great results, extending the lifetime of the patient without riskier surgical interventions. Moreover, liver-directed therapies may clearly benefit a patient who is symptomatic from their tumor or may even allow that patient to be a candidate for surgical treatment in the future. Lastly, nonsurgical, non-liver-directed therapies are considered palliative care in the treatment of NLMs. These systemic therapies are not first line, but can still achieve longer lifespans as a salvage therapy. Newer technologies, including genetic targets such as microRNA subtypes, are fast evolving and will continue to allow patients with NLMs several options. Even though NETs are rare tumors, NLMs are even more rare, and this characteristic prevents large, randomized-controlled trials and modalities of treatment for these tumors continue to improve as there is an obvious need. These treatments are expected to maintain this progression well into the future, especially as knowledge of NLMs increases with additional studies, so that we may provide patients diagnosed with NLMs with every possible chance towards increased survival.
